# A randomised controlled trial of a probiotic ‘functional food’ in the management of irritable bowel syndrome

**DOI:** 10.1186/1471-230X-13-45

**Published:** 2013-03-07

**Authors:** Lesley M Roberts, Deborah McCahon, Roger Holder, Sue Wilson, FD Richard Hobbs

**Affiliations:** 1 Primary Care Clinical Sciences, School of Health and Population Sciences, University of Birmingham, Edgbaston, Birmingham B15 2TT, UK; 2 Department of Primary Care Health Sciences, Oxford University, 2nd Floor, 23-38 Hythe Bridge Street, Oxford OX1 2ET, UK

**Keywords:** Probiotic, Functional food, IBS, RCT

## Abstract

**Background:**

Irritable Bowel Syndrome (IBS) is a common condition characterised by pain, distension and altered bowel habit. Evidence suggests functional foods containing probiotics improve gastrointestinal transit, however, data are limited by short follow-up periods and evaluation in selected populations.

**Methods:**

A multi-centre, randomized, double blind, controlled trial to evaluate the effect of a probiotic vs non-probiotic dairy product on symptoms in IBS with a constipation element (IBS – Constipation or IBS – Mixed profile). Set in 13 general practices within central England. Individuals meeting the ROME III criteria for IBS, aged 18–65 completed a pre-study diary. Eligible individuals were randomized to consume dairy ‘yoghurt’ products which either did or did not contain active probiotics twice daily and to complete a daily diary. Primary outcome was subjective global assessment of symptom relief at week 4. Other outcomes comprised, IBS symptom scores, pain, bloating and flatulence levels, stool frequency, stool consistency, ease of bowel movement and quality of life.

**Results:**

179 were randomized (91 active, 88 placebo). 76 (43 active, 33 placebo) completed the study. No significant between group differences existed at 4 weeks (57% active vs 53% placebo, reported adequate relief (p = 0.71)). By week 8, 46% active vs 68% placebo reported adequate relief (p = 0.03). This was sustained at week 12.

**Conclusions:**

Significant improvements were reported for most outcomes in all trial participants but improvement did not differ by group. This trial does not provide evidence for effectiveness of a probiotic in IBS, in variance with a body of published literature and review conclusions. Differential drop out may however cloud interpretation of data.

UK Trial registration:ISRCTN78863629

## Background

Functional foods are defined as foods which purport to improve health or wellbeing. In 2007 a BMJ editorial called for the closer evaluation of functional foods and recent European Union regulation and directives have formalised the need for such foodstuffs to support claims with robust evidence [1]. Their use by the public has increased considerably, especially in chronic symptomatic disorders with limited treatment options. For example, for the large numbers of individuals suffering symptoms of irritable bowel syndrome (IBS) where conventional treatment is often poorly efficacious, the potential for alternative evidence based therapies is considerable.

IBS, a functional bowel disorder, is a common problem affecting a substantial proportion of the population, with point prevalence estimates ranging from 10-30% [2-10]. It is associated with significantly reduced quality of life [8,11,12] and it is known that many patients seek alternative strategies for symptom relief [13]. One of the simplest and therefore potentially most attractive options to patients is the inclusion of functional foods in the diet, with increasing markets for ‘health drinks’ in the UK [14] and elsewhere.

Among functional foods, probiotics have gained high interest in recent years since gut microbiota may be involved in gastro-intestinal (GI) functions and alterations in gut microbiota have been shown in IBS [15,16]. Recent systematic reviews [17-19] of the literature have evaluated the impact of probiotics on IBS. These reviews have highlighted the considerable heterogeneity in study findings and also in outcomes, probiotic combinations and study design. Therefore, despite the number of studies, the evidence base remains poor with data coming largely from small or uncontrolled trials. Overall, within the published literature and systematic reviews [19-22] there is a suggestion that probiotics confer benefit although limitations of available evidence and possibility of publication bias are acknowledged. Larger well-designed studies of probiotics in IBS are therefore required.

A well investigated fermented milk probiotic product, containing a specific *Bifidobacterium* strain (*Bifidobacterium lactis* CNCM I-2494 (previously DN-173 010)) with lactic acid bacteria (*Lactobacillus delbrueckii* subsp. *bulgaricus* and *Streptococcus thermophilus*), has shown beneficial effects on gut functions in several small randomised controlled studies [23-26]. The consumption of this probiotic dairy product (PDP) was associated with shortening of colonic transit time in patients with IBS with predominant constipation (IBSC) together with improvement of abdominal distension and other GI symptoms [24] and modest reduction of GI discomfort in primary care IBS-C patients [25].

This study aimed to compare improvement in subjective assessment of symptom relief, individual symptoms and quality of life over a 12 week period in individuals consuming this commercially available product (Danone, France) and those consuming an inactive identical product. The study focussed on IBS which included constipation as a feature (either constipation predominant or mixed profile), which accounts for approximately 75% of the IBS population, based on preliminary data on the value of probiotics in this sub group [24].

## Methods

### Design overview

A randomized placebo controlled trial to compare symptom and quality of life in patients with IBS taking a probiotic or non- probiotic fermented dairy product was conducted in primary care practices.

### Setting and participants

13 general practices in the Midlands Research Practices Consortium (MidReC) situated within the West Midlands, UK. Patients were identified through searches of electronic patient records for symptomatic individuals with a diagnosis of IBS > 6 months or having received two or more prescriptions in the previous 24 months for anti-spasmodics, bulking agents or laxatives. A confirmatory screening questionnaire based on ROME III criteria [27] was sent to all patients. Lower than anticipated yield, led to administration of the screening questionnaire to all patients aged 18–65 in five of the thirteen practices. No other alterations were made to the study protocol during trial conduct. General practitioners applied discretion to prevent contact with patients considered inappropriate for contact or inclusion, for example individuals known to be in a terminal stage of illness or experiencing acute and severe mental illness.

Study eligibility was assessed from the information provided on the screening questionnaire plus telephone follow-up to verify responses. Individuals were considered eligible for inclusion if they were aged 18–65, met ROME III [27] criteria for a diagnosis of IBS with symptoms being present for > 6 months and reported a constipation element to their symptom profile (either constipation predominant or a mixed symptom profile).

Eligible patients were mailed a symptom diary for completion over the two weeks prior to their randomization appointment. At the baseline recruitment clinic research nurses confirmed eligibility and applied further study exclusions: individuals reporting passing more than 3 soft stools per day in the absence of laxative mediation during the pre-study phase; individuals without problematic symptoms judged by an IBS Symptom Severity Score (IBS-SSS) [28] of <75; individuals with known organic disease or reporting clinical signs of alarm; individuals who were pregnant, breast-feeding or lactose intolerant; individuals with a BMI >35 or <18, as this was deemed suggestive of abnormal diet or function; and those with a recent change of IBS medication (in the preceding month) to reduce likelihood of co-intervention bias.

### Randomization and interventions

Blocked randomization lists (1:1 allocation) were produced by the lead statistician (RH) for each general practice site and held centrally. Because of the short shelf-life of dairy products randomised allocation lists were sent to the sponsors for product production prior to randomisation clinics so that two weeks supply of the relevant allocated product was available at the point of randomization. All volunteers gave written informed consent before inclusion in the study.

The test product was a commercially available fermented product containing *Bifidobacterium lactis* (strain number I-2494 in French National Collection of Cultures of Micro-organisms (CNCM, Paris, France; Danone collection number DN-173 010) together with the two classical yoghurt starters, *S. thermophilus* (CNCM strain number I-1630) and *L. bulgaricus* (CNCM strain numbers I-1632 and I-1519). The test product contains 1.25×10^10^ colony forming unit (cfu) of *Bifidobacterium lactis* CNCM I-2494 per cup and 1.2×10^9^ cfu/cup of *S. thermophilus* and *L. bulgaricus*.

The control product was a milk-based non-fermented dairy product without probiotics and with similar lactose content to the test product. Both the test and control products were without flavour. Each pot contained 125 g. Both products were specifically prepared for the study and provided by Danone Research (Palaiseau, France). Nurses, GPs, patients and the research team remained blind to product allocation until all analyses had been completed.

In both groups consumption of the product twice daily was instructed and all patients received a two week supply of the allocated product in the first instance and instructions to collect repeat supplies fortnightly for 12 weeks.

### Outcomes and follow-up

All patients returned completed daily diaries at follow-up visits after 4, 8 and 12 weeks. The daily diary assessed stool frequency and consistency (evaluated in accordance with the Bristol stool scale) and bowel movement difficulty (using a 5-point Likert scale which ranged from no difficulty to extreme difficulty). Weekly entries assessed subjective global assessment (SGA) [29,30] of symptom relief (“Do you consider that in the past week you have had adequate relief of your IBS symptoms?”) and individual IBS specific symptom assessment of bloating, pain and flatulence (6 point Likert scale ranging from 0 (none) to 5 (very severe)).

Participants completed the Birmingham IBS Symptom Score [31], the IBS SSS [27] and an IBS specific quality of life (QoL) tool [32] at baseline and at weeks 4, 8 and 12. The Birmingham Symptom Score [31] uses 11 questions to assess pain, diarrhoea and constipation, scores range from 0–100 for each dimension with higher scores indicating greater well-being. The IBS –SSS [27] utilises five questions each generating a maximum score of 100 and a total possible score of 500 with higher scores indicating a greater symptom burden. The IBS specific QoL [32] tool evaluates dimensions; dysphoria, interference with activity, body image, health worry, food avoidance, social reaction, sexual and relationship using 34 five point Likert statements. Items are summed to create a total score with higher scores indicating better QoL.

The primary outcome was pre-specified as SGA of symptom relief at week 4 in line with the recent trial evidence on which the study was powered [33] and after discussion with the product manufacturer and sponsor. Comparison of all other outcomes at weeks 4, 8 and 12 between groups formed specified secondary outcome measures.

### Statistical analysis

The sample size calculation was based on a previous study [33] demonstrating a 20% between group difference (62% active vs. 42% control) for SGA. Therefore, 107 patients with IBS-C were required in each group to demonstrate a 20% difference between groups in proportions reporting adequate IBS symptoms relief at week 4, with a significance of 5% and 80% power. This was inflated to 240 patients to be divided in two groups of 120 to allow for drop-outs. No stopping guidelines were in place due to the short trial duration and assumed safety of a commercially available product. Data analyses for all outcomes were performed on an intention to treat basis (ITT) and further analyses were undertaken to compare differences in those not deviating from protocol (per protocol). Analyses were performed on available data for each parameter. ANCOVA with primary care centre as a random effect, baseline as a covariate and product as a fixed effect was used to compare the intervention and control groups with respect to all outcomes. Ordinal logistic regression with centre and product as fixed effects was used to explore change within each arm of the study. Analyses were conducted using MINITAB version 14, S Plus version 8 and ML wiN version 2.1 statistical software.

### Role of the funding source

The study was funded by Danone Ltd. Contracts including a right to publication of all findings were approved and signed by both parties prior to commencement of the research. The funders contributed to discussion about study design and selection of outcome measures. All subsequent data review was blinded and the analysis was undertaken independently of the funders. The paper was reviewed by the sponsor and an expert nominated by them with some requested changes included in the final version.

## Results

**Figure 1:**
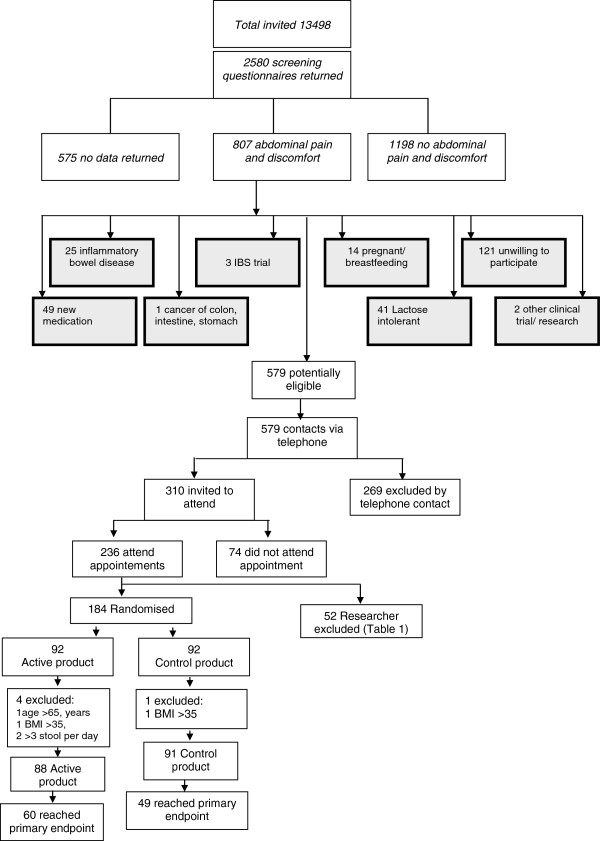
CONSORT diagram of recruitment, randomization & primary endpoint numbers.

In summary, of 13,498 individuals screened by questionnaire, 2580 responded. (Figure 1) Of these, 807 reported the experience of abdominal pain in the preceding 3 months and 579 were deemed potentially eligible. Telephone screening excluded a further 269 individuals, primarily because of a diarrhoea predominant form of IBS, and 310 individuals were issued with a clinic appointment, of which 236 attended. A further 52 were excluded for reasons described in Table 1. One hundred and eighty four individuals were randomized although 5 were subsequently excluded when data quality checks revealed breached exclusion criteria. Eighty eight individuals commenced consumption of the active product and 91 of the control product leading to an ITT population of 179 patients.

**Table 1:** Exclusions made during randomisation clinics

**Exclusion**	**n = 52 (%)**
Average baseline stools frequency >3	14 (27%)
Does not wish to participate	13 (25%)
BMI too high	9 (17%)
Pre study diary not fully completed	3 (6%)
Clinical signs of alarm	3 (6%)
BMI too low	1 (2%)
Anti-psychotic medication in last month	1 (2%)
Lactose intolerance or immunodeficiency	1 (2%)
Organic bowel disease	1 (2%)
2 week holiday during next 4 weeks	1 (2%)
Change of treatment for IBS in last 4 weeks	1 (2%)
History of laxative abuse	1 (2%)
Diet change in last 4 weeks	1 (2%)
IBS SSS less than 75	1 (2%)
General anaesthesia in last month	1 (2%)

Groups were similar with respect to baseline IBS symptom severity scores, demographics, medical history and concomitant disease, with the exception of mental illness with 9 individuals in the active group having a current mental health diagnosis compared to 1 in the control group (Table 2).

**Table 2:** Baseline demographic data and symptom scores of participants

	**Active product**	**Control product**
	**n = 88**	**n = 91**
	**Mean (SD)**	**Mean (SD)**
Age	44.66 (11.98)	43.71 (12.76)
BMI	26.75 (4.03)	25.85 (3.61)
Percentage		
Male	15	15
Non-smoker	47	56
Light smoker (<10 per day)	8	6
Heavy smoker (>10 per day)	14	18
Former smoker	32	21
White ethnic group	91	86
Other ethnic group combined	9	14
Mental Health	10	1
Score		
*IBS-SSS (Max score 500 – higher scores indicating greater symptom burden*	258.70 (86.51)	251.73 (76.13)
*IBS symptoms (Birmingham Score) (Max score 100 for all dimensions – higher scores represent greater well-being)*		
Constipation	47.31 (26.86)	49.08 (25.75)
Diarrhoea	84.58 (15.17)	85.62 (11.72)
Pain	62.50 (19.74)	60.95 (19.62)
Total	68.36 (12.00)	68.90 (11.07)
*IBS specific symptoms (Scored 0–5 with greater scores indicating greater symptom burden)*		
Pain	2.50 (1.08)	2.50 (1.08)
Bloating	3.13 (1.18)	3.16 (0.97)
Flatulence	3.18 (1.14)	2.88 (1.16)
Composite score	8.80 (2.65)	8.54 (2.50)
*Stool frequency (Stools per day)*	1.17 (0.93)	1.19 (0.96)
*Stool consistency (From Bristol Stool Form)*	3.14 (1.64)	3.20 (1.60)
Bowel movement difficulty *(Scored 0–5 with higher scores indicating greater difficulty)*	1.38 (1.19)	1.18 (1.06)
*IBS-QoL (Multi-item scale with greater scores indicating better quality of life)*		
Dysphoria	66.69 (27.72)	72.94 (23.49)
Activity interference	73.02 (23.06)	73.73 (21.12)
Body image	58.03 (26.07)	60.85 (23.60)
Health worry	56.77 (26.13)	64.65 (24.66)
Food avoidance	58.14 (28.53)	59.34 (30.55)
Social reaction	64.99 (26.08)	73.72 (24.34)
Sexual	75.43 (31.85)	77.75 (26.72)
Relationship	75.43 (24.06)	80.86 (20.25)
Total score	66.45 (22.49)	70.78 (20.08)

### Primary outcome

Among the 179 randomized patients, 109 had available data for SGA at week 4 (60 in active group; 49 in control group). There were no between group differences in SGA noted at week 4 with 56.7% of the active group and 53.1% of the control group reporting adequate symptom relief (p = 0.71) (Table 3).

**Table 3:** Comparison of the proportions reporting adequate symptom relief in intention to treat analyses

**Subjective global assessment (SGA)**	**Active product % (n)**	**Control product % (n)**	**Difference between the groups**	**p value active vs control**
**Week 4**	56.7 (60)	53.1 (49)	3.61	0.707
**Week 8**	46.2 (52)	68.3 (41)	−22.1	0.027
**Week 12**	45.8 (48)	75.8 (33)	−29.9	0.004

Large numbers of withdrawals accrued, with dropout rates of 39% (70/179) at week 4, 48% (86/179) at week 8 and 55% (98/179) at week 12, despite efforts to follow-up all participants. Withdrawal reasons were recorded where provided by participants. Across both groups drop-out was attributable to a range of factors including dislike of product taste, changes in personal circumstances and reported adverse events such as nausea.

At week 8 greater benefit in terms of adequate symptom relief was reported in the control group (68.3% reporting relief versus 46.2%, p = 0.03) and this was sustained at week 12 (75.8% versus 45.8%, p = 0.004) (Table 3).

### Secondary outcomes

During the initial 4 weeks of the trial statistically significant (at the p < 0.05 level) improvement from baseline was reported in both groups for the majority of outcomes, including improvements in symptoms scores, bloating, flatulence, ease of bowel movement and quality of life (Table 4). Although both groups reported improvement there was no significant between group difference when considering changes in either of the symptom measures used, nor for any individual symptoms or QoL outcomes (Table 4). The improvement from baseline scores demonstrated at week 4 in both groups remained significant for most outcomes at week 8. The absence of a between group difference, however remained constant with both groups demonstrating similar improvements (Table 5).

**Table 4:** Between and within group comparisons at week 4

**Study parameter**	**Active product**			**Control product**			**Active versus control**
	**n**	**Mean (week 4–baseline) (SD)**	p value* (change from baseline)	**n**	**Mean (week 4–baseline) (SD)**	p value* (change from baseline)	p value**
**IBS-SSS**	41	−53.35 (81.98)	<0.002	44	−31.11 (78.57)	0.010	0.233
**Birmingham IBS symptom scale**							
Constipation	59	9.44 (20.40)	<0.001	49	15.10 (21.59)	<0.001	0.131
Diarrhoea	59	3.88 (12.26)	0.007		2.22 (10.17)	0.106	0.616
Pain	60	7.89 (14.70)	<0.001		11.22 (18.34)	<0.001	0.367
Total score	60	6.10 (10.83)	<0.001		8.28 (11.10)	<0.001	0.251
**IBS specific symptoms**							
Pain	60	−0.33 (1.19)	0.087	49	−0.61 (1.36)	0.001	0.109
Bloating		−0.66 (1.35)	<0.001	48	−0.68 (1.12)	<0.001	0.432
Flatulence		−0.51 (1.24)	<0.001	49	−0.36 (1.07)	0.0017	0.955
Composite score		−1.48 (3.03)	0.002	49	−1.69 (2.99)	<0.001	0.264
Stool frequency	417	0.07 (0.89)	0.202	341	0.04 (0.85)	0.607	0.648
Stool consistency	341	0.13 (1.20)	0.240	269	0.31 (1.30)	0.007	0.184
Bowel movement difficulty	341	−0.24 (0.97)	0.004	271	−0.28 (0.89)	0.011	0.450
**IBS Quality of life**							
Dysphoria	60	9.78 (16.89)	<0.001	50	7.53 (15.54)	0.002	0.875
Activity interference	60	5.68 (10.97)	0.003	50	5.07 (13.77)	0.008	0.769
Body image	60	6.63 (16.28)	<0.001	50	8.13 (17.37)	0.004	0.134
Health worry	60	7.92 (15.20)	0.001	50	5.75 (16.17)	0.009	0.958
Food avoidance	60	9.58 (16.01)	0.002	50	4.42 (18.76)	0.078	0.121
Social reaction	60	7.33 (14.23)	0.003	50	4.17 (14.10)	0.029	0.769
Sexual	59	4.24 (15.68)	0.171	48	4.17 (20.36)	0.125	0.853
Relationship	60	7.15 (15.54)	<0.001	50	1.92 (12.43)	0.217	0.139
Total score	60	7.60 (10.88)	<0.001	50	5.69 (12.60)	0.004	0.979

*ANCOVA with centre as a random effect, baseline as a covariate and product as a fixed effect.

**Ordinal logistic regression with centre and product fixed effects.

Stool frequency, consistency and bowel movement difficulty were recorded daily and therefore n represents the number of data items (days) included in analysis for these items.

**Table 5:** Between and within group comparisons at weeks 8 and 12

**WEEK 8**							
**Study parameter**	**A = Active product**			**C = Control product**			**A vs C**
	**n**	**Mean (week 12–baseline) (SD)**	p value* (change from baseline)	**n**	**Mean (week 12–baseline) (SD)**	p value* (change from baseline)	p value**
**IBS-SSS**	50	−58.30 (84.96)	<0.001	40	−58.33 (73.37)	<0.001	0.589
**Birmingham IBS symptom scale**							
Constipation	52	13.78 (24.47)	<0.001	41	17.24 (22.61)	<0.001	0.302
Diarrhoea		4.25 (13.78)	0.210		2.15 (7.71)	0.064	0.907
Pain		11.28 (18.88)	<0.001		13.66 (16.80)	<0.001	0.257
Total score		8.71 (13.09)	<0.001		9.44 (9.75)	<0.001	0.265
**IBS specific symptoms**							
Pain	53	−0.58 (1.24)	0.012	41	−0.66 (1.43)	0.002	0.292
Bloating	52	−0.80 (1.44)	0.005		−0.85 (1.26)	<0.001	0.408
Flatulence	53	−0.54 (1.28)	0.002	40	−0.50 (1.08)	0.004	0.864
Composite score	52	−1.95 (3.43)	0.003		−1.96 (3.17)	<0.001	0.374
Stool frequency	346	0.07 (0.95)	0.303	276	0.10 (0.05)	0.141	0.828
Stool consistency	290	0.18 (1.17)	0.042	218	0.50 (1.23)	0.001	0.098
Bowel movement difficulty	291	−0.43 (1.01)	<0.001	224	−0.40 (0.78)	<0.001	0.623
**IBS Quality of life**							
Dysphoria	53	14.66 (19.87)	<0.001	41	9.29 (13.95)	<0.001	0.508
Activity interference		8.20 (14.74)	0.001		6.05 (12.99)	0.001	0.924
Body image		11.95 (15.98)	<0.001		8.54 (13.46)	<0.001	0.735
Health worry		13.29 (17.81)	<0.001		7.62 (15.19)	0.002	0.428
Food avoidance		10.06 (17.63)	<0.001		5.49 (17.84)	0.039	0.372
Social reaction		10.85 (16.18)	<0.001		6.71 (12.21)	<0.001	0.462
Sexual		8.41 (18.31)	0.006		7.05 (16.92)	0.005	0.554
Relationship		7.23 (18.64)	0.015		7.01 (9.88)	<0.001	0.635
Total score		10.95 (13.14)	<0.001		7.52 (10.22)	<0.001	0.483
**WEEK 12**							
**IBS-SSS**	43	−61.02 (75.78)	<0.001	33	−97.24 (90.17)	<0.001	0.028
**Birmingham IBS symptom scale**							
Constipation	48	6.65 (22.56)	0.039	35	17.52 (23.60)	<0.001	0.011
Diarrhoea		3.27 (11.17)	0.048		2.43 (10.60)	0.133	0.889
Pain		10.28 (14.09)	<0.001		15.24 (17.38)	<0.001	<0.107
Total score		6.07 (9.62)	<0.001		10.10 (10.78)	<0.001	<0.022
**IBS specific symptoms**							
Pain	48	−0.59 (1.24)	0.002	34	−1.04 (1.33)	<0.001	0.016
Bloating	48	−0.58 (1.39)	0.005		−1.35 (1.21)	<0.001	0.006
Flatulence	48	−0.64 (1.04)	0.001		−0.76 (1.23)	<0.001	0.383
Composite score	48	−1.81 (3.03)	<0.001		−3.16 (3.05)	<0.001	0.011
Stool frequency	311	−0.07 (0.75)	0.186	224	0.07 (0.82)	0.464	0.211
Stool consistency	247	0.26 (1.22)	0.045	181	0.31 (0.99)	0.012	0.784
Bowel movement difficulty	246	−0.35 (0.99)	<0.001	180	−0.36 (0.86)	<0.001	0.329
**IBS Quality of life**							
Dysphoria	48	13.61 (20.12)	<0.001	34	10.83 (10.68)	<0.001	0.933
Activity interference		8.47 (12.31)	<0.001	34	7.09 (8.73)	<0.001	0.806
Body image		11.33 (14.16)	<0.001	34	8.27 (14.08)	0.005	0.857
Health worry		14.15 (21.01)	<0.001	34	9.07 (13.19)	<0.001	0.634
Food avoidance		8.16 (20.45)	0.008	34	8.58 (13.37)	<0.001	0.687
Social reaction		9.64 (17.15)	<0.001	34	9.38 (8.74)	<0.001	0.660
Sexual		8.07 (21.34)	0.031	32	10.55 (16.53)	<0.001	0.382
Relationship		7.03 (15.81)	0.002	34	5.51 (9.22)	<0.001	0.751
Total score		10.43 (12.92)	<0.001	34	8.83 (6.12)	<0.001	0.810

*ANCOVA with centre as a random effect, baseline as a covariate and product as a fixed effect.

**Ordinal logistic regression with centre and product fixed effects.

Stool frequency, consistency and bowel movement difficulty were recorded daily and therefore n represents the number of data items (days) included in analysis for these items.

By week 12, however, change in scores differed significantly between groups for the following outcomes; IBS symptom severity score, IBS symptoms of constipation and associated total score as measured by the Birmingham score, IBS specific symptoms of pain, bloating and associated total score. For all statistically significant outcomes those receiving the control product demonstrated greater improvement than those receiving the active product (Table 5).

Per protocol analyses undertaken at each of the time points (4, 8 and 12 weeks) did not significantly alter the findings reported in the ITT analyses.

## Discussion

### Summary of main findings

In this double-blind randomized controlled trial of a commercially available probiotic fermented dairy product (yoghurt) containing *bifidobacterium animalis* DN-173010 in community based IBS patients with a constipation element, both the inactive (control) and active product groups demonstrated significant improvement across a range of symptom and quality of life outcomes. Improvement was maintained throughout the 12-week period of observation. Reductions in symptoms were, however, reported at similar levels in both groups when assessed in weeks 4 and 8. At week 12 there was the suggestion of a benefit of the control product over the active formulation but this is likely to be attributable to differential drop-outs between groups.

The sustained and large improvement observed in both groups suggests there may be benefit from regular consumption of a dairy product but does not suggest any additional benefit of the addition of a probiotic to such products. If such a benefit is accrued the mechanism through which this works is unclear and product effect, regularisation of eating habits and increased fluid intake are all possible explanations. The fact that benefits were also accrued by control participants supports these potential mechanisms of effect. In the absence of a non-intervention group this effect cannot be confirmed but the substantial effect size is worthy of further consideration and exploration of sub-groups in which greatest benefit is accrued would be advisable. In this study there was the suggestion that drop-out may be related to baseline symptom severity with those remaining in the study having greater baseline symptom severity scores than those withdrawing (IBS SSS 264.15 versus 238.09, p = 0.04). This could suggest greater benefit was accrued by those with more severe symptoms encouraging continued participation or may reflect and greater motivation to pursue therapy in this group.

### Study strengths and limitations

This primary care based RCT is one of the largest trials of probiotics in IBS to date and one of the only functional food trials to be conducted outside small hospital populations. The main study limitation was not reaching the target recruitment of 240 patients between the two arms, with only 179 recruited of whom 109 provided data for the primary outcome. The failure to meet the trial target recruitment was due to a contractually fixed end date for the trial and the 109 participants providing 4 week data reduced study power to 47%. However, at study close the trial showed futility of continuance given the observed lack of treatment benefit. A post-hoc re-estimate of sample size, based on observed difference in the study primary outcome (proportions 0.57 versus 0.53), indicated 2477 patients would have been required in each arm to demonstrate a statistically significant treatment effect.

The observed imbalance in the randomization numbers was due to the study requirement to randomize eligible patients prior to the baseline visit (to enable product manufacture) and there was differential clinic attendance by chance. Since the study power was reduced through a high level of drop-outs and differential drop-out, it therefore remains a possibility that an effect of the active product was missed. The high drop-out rate highlights the difficulty of conducting functional food trials but does call into question the acceptability of such interventions to patients over even short periods.

### Comparison with existing literature

This study focused only on individuals who had a constipation element to their IBS since earlier data suggested this might be the population who might obtain most benefit from consumption of a probiotic: prior trials [34-36] have suggested that daily consumption of fermented milk products containing Bifidobacterium animalis DN-173 010 improves transit and alleviates bloating in individuals with low stool frequency. However the heterogeneous nature of the cohort recruited (some with a constipation predominant form of IBS and others with a mixed profile) may further mask an effect which would be demonstrated in a more homogeneous IBS constipation predominant patient group. Despite screening to ensure all participants were symptomatic at baseline and with a constipation feature to their IBS, the mean number of stools passed daily at baseline was greater than one and the mean stool consistency was 3 on the Bristol Stool Form Scale which equates to only a slightly drier than average stool suggesting this cohort did not comprise those with more significant forms of constipation. Recruitment of participants with a more extreme manifestation of constipation may have more closely replicated the small hospital populations in previous trials where modest benefits of probiotics were observed. However, given the dominance of small positive trials in the literature, it is also possible that the literature in this area is subject to publication bias. This highlights the importance of establishing the efficacy of treatments across the wide range of IBS phenotypes if they are to be made widely available. If functional foods are to be advocated for a common disorder like IBS it is important to have clear indications as to the symptoms most likely to accrue benefit. This study importantly illustrates that data from the presumed more persistent and extreme IBS phenotypes seen in hospitals cannot routinely be extrapolated to the general population.

The interest in the use of functional foods is demonstrated by the large numbers of reviews conducted in recent years [20,21,37-41]. Despite this major body of review evidence, the trial evidence upon which it is based remains limited. This carefully conducted double blind randomized 12 week study assessing a wider range of outcomes in a general population of IBS with a constipation element, did not reproduce these earlier hospital based findings. Explanations for this may be attributable to differences in participant groups, outcomes and follow-up. However this study best reflects current practice for the majority of patients with IBS who might consider probiotic milk products to try to reduce symptoms.

### Implication for future research or clinical practice

Clinicians advising patients with IBS managed in the community featuring a constipation element may wish to suggest the inclusion of a fermented dairy product, given that significant improvements were reported for most outcomes in all trial participants. The requirement of such products to contain a probiotic is not supported by this study.

Further research is required to consider the mechanism via which improvement in symptoms may be effected in trials of this nature through consideration of dietary habits and fluid intake. Such work will ensure the most accurate dietary advice can be provided to patients.

## Conclusions

Significant improvements were reported for most outcomes in all trial participants but improvement did not differ by intervention or placebo group. This trial therefore does not provide evidence for effectiveness of a probiotic in IBS, in variance with much published literature and review conclusions.

## Ethical approval

Ethical approval was obtained from Nottingham Research Ethics Committee 2 ref 06/Q2404/172 prior to commencement of the study.

## Competing interests

All authors declare that:

1. DM and RH salary or supervision costs were funded in part from a research grant received from Danone Ltd for the conduct of this study. Payments received were exclusively used by the University of Birmingham to offset the salary costs of these individuals for their time on the study. All surpluses were retained by the University.

2. All authors confirm no other relationship with Danone Ltd.

3. A publication clause was included within the contract between Danone Ltd and the University of Birmingham permitting publication of all findings after Danone had 2 months notice to comment on the final draft.

4. All authors confirm their spouses, partners and children have no financial relationships which may be relevant to the submitted work.

5. All authors confirm that they have no non-financial interest that may be relevant to the submitted work.

## Authors’ contributions

The study was designed by LR, SW and FDRH following an approach from Danone Ltd. LR and DM undertook management of the study, including overseeing data collection and management and quality assurance. RH undertook all analyses. All authors contributed to data interpretation. LR wrote the first draft of this paper and all authors were responsible for subsequent critical revision of the manuscript. FDRH is the guarantor and corresponding author for this paper. All authors read and approved the final manuscript.

## Pre-publication history

The pre-publication history for this paper can be accessed here:

http://www.biomedcentral.com/1471-230X/13/45/prepub
